# Using classification tree modelling to investigate drug prescription practices at health facilities in rural Tanzania

**DOI:** 10.1186/1475-2875-11-311

**Published:** 2012-09-05

**Authors:** Dan K Kajungu, Majige Selemani, Irene Masanja, Amuri Baraka, Mustafa Njozi, Rashid Khatib, Alexander N Dodoo, Fred Binka, Jean Macq, Umberto D’Alessandro, Niko Speybroeck

**Affiliations:** 1 INDEPTH Network, P.O Box KD 213 Kanda, Accra, Ghana; 2 Ifakara Health Institute, PO Box 78373, Dar es Salaam, Tanzania; 3 Centre for Tropical clinical Pharmacology #38; Therapeutics, University of Ghana Medical School, P.O Box KB4236, Accra, Ghana; 4 Université Catholique de Louvain, Belgium, Clos Chapelle-aux Champs, Bruxelles 1200, Belgium; 5 Medical Research Council Unit, The Gambia, P.O Box 273, Banjul, The Gambia and Institute of Tropical Medicine, Antwerp, Belgium

**Keywords:** Polypharmacy, Co-prescription, Anti-malarials, Classification trees, Data mining, Tanzania

## Abstract

**Background:**

Drug prescription practices depend on several factors related to the patient, health worker and health facilities. A better understanding of the factors influencing prescription patterns is essential to develop strategies to mitigate the negative consequences associated with poor practices in both the public and private sectors.

**Methods:**

A cross-sectional study was conducted in rural Tanzania among patients attending health facilities, and health workers. Patients, health workers and health facilities-related factors with the potential to influence drug prescription patterns were used to build a model of key predictors. Standard data mining methodology of classification tree analysis was used to define the importance of the different factors on prescription patterns.

**Results:**

This analysis included 1,470 patients and 71 health workers practicing in 30 health facilities. Patients were mostly treated in dispensaries. Twenty two variables were used to construct two classification tree models: one for polypharmacy (prescription of ≥3 drugs) on a single clinic visit and one for co-prescription of artemether-lumefantrine (AL) with antibiotics. The most important predictor of polypharmacy was the diagnosis of several illnesses. Polypharmacy was also associated with little or no supervision of the health workers, administration of AL and private facilities. Co-prescription of AL with antibiotics was more frequent in children under five years of age and the other important predictors were transmission season, mode of diagnosis and the location of the health facility.

**Conclusion:**

**S**tandard data mining methodology is an easy-to-implement analytical approach that can be useful for decision-making. Polypharmacy is mainly due to the diagnosis of multiple illnesses.

## Background

Irrational drug use or misuse means the distribution or consumption of drugs in ways that negate or reduce their efficacy or in situations where they are unlikely to have the desired effect [1]. Besides being an economic burden for the patients, communities and health systems, it may result in drug resistance, ineffective treatment and adverse drug events. According to the World Health Organization (WHO) the ‘rational use of drugs requires that patients receive medications appropriate to their clinical needs, in doses that meet their own individual requirements for an adequate period of time, and at the lowest cost to them and their community’ [2]. Irrational drug use is a widespread practice in developing counties [3], though it occurs in high-income countries, e g, antibiotics [4,5].

Tanzania revised its malaria treatment policy in 2006 and replaced sulphadoxine-pyrimethamine (SP) with artemether-lumefantrine (AL) as first-line treatment for uncomplicated malaria [6], complying with the WHO guidelines [7]. Such policy change was necessary because of the decreasing efficacy of SP [6]. The success of a new treatment policy partly depends on the compliance of prescribers’ to the national guidelines and on the patients’ adherence to the treatment [8]. Irrational treatment practices are more common in the private than in the public sector and in most developing countries the private sector manages over half of all malaria cases [9]. Prompt and effective treatment of malaria patients is one of the cornerstones of the global efforts to reduce malaria morbidity and mortality. As many countries in sub-Saharan Africa are introducing relatively expensive artemisinin-based combination therapy (ACT) into the formal health system for uncomplicated malaria, policy makers, programme managers and donors are interested in assessing the quality of malaria case management with ACT, in particular ensuring that malaria patients are appropriately treated with an ACT [10].

The INDEPTH effectiveness and safety studies of anti-malarial drugs in Africa (INESS) project is a Phase IV study on both existing and new combination anti-malarial therapies in at least seven INDEPTH demographic surveillance system (DSS) sites in four African countries. This project effectively creates the missing final section of the drug development pipeline for Africa by ensuring local evidence on treatment effectiveness. The purpose is to minimize the time gap between licensure and adoption of new anti-malarials by providing objective endemic-country effectiveness data that will help inform global and national policy and practice. This project also enhances the capacity of Africa to monitor local health systems costs, effective coverage, and effects of new or alternative post-registration anti-malarial treatments.

The health facility survey conducted as part of the evaluation of system effectiveness of ACT, generated data on drug prescriptions from patients and health workers in rural health facilities, either private or public. This analysis besides describing the drug prescription patterns illustrates the use of standard data mining methods of classification tree analyses for investigating the influence specific patient, health care provider or health facility characteristics may have on drug prescription.

## Methods

### Study area and population

The study was conducted in March and October 2010 on patients and health workers in health facilities located within the Rufiji and Ifakara health and demographic surveillance system (HDSS) areas. Rufiji district in the coast region has about 182,000 inhabitants and a HDSS in place since 1998, covering a population of about 85,000 inhabitants [11]. The Ifakara HDSS is located in southern Tanzania and covers two districts (Kilombero and Ulanga) in Morogoro region, with a population of about 99,000 people [12]. All government and non-government health facilities providing outpatient care within the HDSS areas were included (16 in Rufiji and 14 in Ifakara). Investigators visited each facility for two to three days and collected information on attending patients. The target sample size was 720 patients per HDSS to estimate the proportion of those with uncomplicated malaria correctly treated with an ACT with 10% precision, assuming these represented 20% of all patients, that 75% of malaria patients would be treated with an ACT, and a design effect of 2. All patients attending for illness the health facilities on the days of the survey were eligible. Health workers were following the national guidelines for diagnosis and treatment of malaria.

### Data collection

After providing their consent to be included in the survey, patients were interviewed prior to leaving the health facility using standardized questionnaires developed in English and translated into Kiswahili. Information on history of fever, health worker’s diagnoses, laboratory tests, medications prescribed and counselling messages was collected. Pregnant patients were excluded from the survey. Health workers providing outpatient consultations were also interviewed on pre-service training, work experience, in-service training and supervision visits received. Assessment of the level of staffing, availability of diagnostics, medications and other medical supplies was done at the health facility. A list of variables used in the analysis is shown in Table 1.

**Table 1:** Classification tree variables

**Type**	**Variable**	**Levels**
Health facility-related	Type of health facility	Hospital
		Health Centre
		Dispensary
	Ownership of health facility	Public
		Private
	Any artemether lumefantrine available in health facility today	No
		Yes
	Supplies to administer oral medications available	No
		Yes
	Availability of any scale in the health facility	No
		Yes
	No stock-outs of artemether lumefantrine in previous 90 days	No
		Yes
Health worker-related	Mode of diagnosis	Laboratory confirm
		Presumptive diagnosis
	Health workers' cadre	Medical Officer/ Doctor
		Medical Assistant/ Clinical Officer
		Nurse and lower level cadres
	Health worker sex	Male
		Female
	Health worker age	*Continuous variable*
	Patient seen by health worker with medical training	No
		Yes
	Possession of national malaria guideline	No
		Yes
	Possession of national malaria wall chart	No
		Yes
Patient- related	Total number of diagnoses presented on a single visit	*Continuous variable*
	Age group of the patients	Less than 5 yrs
		5-14 yrs
		15 yrs and above
	Patient treated with artemether lumefantrine	No
		Yes
	Patient sex	Male
		Female
	Transmission season	High season
		Low season
	Patient seen by health worker who trained on use of new anti-malarial	No
		Yes
	Patient seen by experienced health worker	No
		Yes
	Patient seen by health worker supervised within previous six months	No
		Yes
	Patient seen by health worker with IMCI training	No
		Yes

### Data management and analysis

Data were double entered and validated using EpiData 3.1 [13], cleaned, managed and analysed using STATA 11[14] and SPSS [15]. Measures for central tendency and dispersion used to describe these data were mean and 95% confidence intervals for continuous variables and percentages for categorical variables. The population was first described on demographics and other variables of importance.

This paper describes and analyses the INESS project health facility survey data from Tanzania. The different methods of data analysis applied in this paper are explained in the next section, starting from standard data mining techniques to investigate the predictors of polypharmacy and co-prescription of AL with an antibiotic at health facilities in rural Tanzania. Polypharmacy is defined as a practice where a patient was prescribed three or more medications at a single encounter in a health facility or with a health worker.

### Statistical methodology

Logistic regression is the standard technique used to investigate the relationship between binary response variables and a set of explanatory variables. However, with the large number of explanatory variables involved, because of potential co-linearity and interactions, it would be complex to investigate for each covariate the nature of the relationship (linear, interaction terms, quadratic, etc.). Moreover, with 22 variables there are 462 possible two-way interactions and it is not feasible to consider all of them. For these reasons, non-parametric classification tree modelling methodologies were used [16-18]. A classification and regression tree (CART) analysis is a useful nonparametric data-mining technique. This analysis is particularly helpful when attempting to investigate which direct and indirect measures of risk are predictive of a newly emerging or complex disease [17]. Contrary to classical regression that uses linear combinations, this method does not require the data to be linear or additive. Furthermore, classification tree analysis does not require to pre-define possible interactions between factors [18]. Therefore, the resulting classification trees accommodate in an intuitive manner more flexible relationships among variables, missing covariate values, multi-colinearity, and outliers [19]. When values for some predictive factors are missing, they can be estimated using other predictor (“surrogate”) variables, permitting the use of incomplete data sets when generating regression trees [16,18]. Another advantage of classification tree analysis (compared with a classical multivariate regression analysis) is that it allows for the calculation of the overall discriminatory power, or relative importance, of each explanatory variable.

### Classification tree modelling

To gain more insight into factors related to dependant variables of polypharmacy (prescription of ≥3 drugs) and co-prescription of AL with antibiotics in rural health facilities, the binary classification tree modelling methodology as introduced by Breiman [18] was used. Classification trees are widely used in applied fields as diverse as medicine (diagnosis), computer science (data structures), botany (classification), and psychology (decision theory) [18-20]. Classification trees are popular in applied fields partly because they are agreeable to graphical display and easy to interpret compared to the use of strict numerical interpretation. Flexibility and hierarchical nature are two important features characterizing classification trees.

Classification tree analysis is a nonlinear and nonparametric model that is fitted by binary recursive partitioning of multidimensional covariate space. The analysis successively splits the data set into increasingly homogeneous subsets until it is stratified to meet specified criteria [16,18,19,21,22]. The Gini index was used as the splitting method, and 10-fold cross-validation was used to test the predictive capacity of the obtained trees. The classification tree method performs cross validation by growing maximal trees on subsets of data then calculating error rates based on unused portions of the data set. To accomplish this, it divides the data set into 10 randomly selected and roughly equal “parts,” with each part containing a similar distribution of data from the populations of interest (e.g., polypharmacy *vs* single prescription). The method then uses the first nine parts of the data, constructs the largest possible tree, and uses the remaining 1/10 of the data to obtain initial estimates of the error rate of the selected sub-tree. The process is repeated using different combinations of the remaining nine subsets of data and a different 1/10 data subset to test the resulting tree. This process is repeated until each 1/10 subset of the data has been used as to test a tree that was grown using a 9/10 data subset. The results of the 10 minitests are then combined to calculate error rates for trees of each possible size; these error rates are applied to prune the tree grown using the entire data set. The consequence of this complex process is a set of fairly reliable estimates of the independent predictive accuracy of the tree, even when some of the data for independent variables are incomplete, specific events are either rare or overwhelmingly frequent, or both.

For each node in a generated tree, the “primary splitter” is the variable that best splits the node, maximizing the purity of the resulting nodes. When the primary splitting variable is missing for an individual observation that observation is not discarded but, instead, a surrogate splitting variable is sought. A surrogate splitter is a variable whose pattern within the data set, relative to the outcome variable, is similar to the primary splitter. Thus, the program uses the best available information in the face of missing values. In data sets of reasonable quality, this allows all observations to be used. This is a significant advantage over more traditional multivariate regression modeling, in which observations missing any of the predictor variables are often discarded [17-19].

### Variable relative importance ranking

One of the goals of classification tree analysis is to develop a simple tree structure for predicting data, resulting in relatively few variables that appear explicitly as splitters, a result that may suggest that the other variables are not important in understanding or predicting the dependent variable. However, unlike a linear regression model, a variable in a classification tree modelling can be considered highly important even if it never appears as a node splitter [19,21,23,24]. Because the method keeps track of ‘surrogate’ splits in the tree-growing process, the contribution a variable can make in prediction is not determined only by primary splits.

To calculate a variable importance score, the classification tree analysis method looks at the improvement measure attributable to each variable in its role as either a primary or a surrogate splitter. The values of ALL these improvements are summed over each node and totalled, and are then scaled relative to the best performing variable. The variable with the highest sum of improvements is scored 100, and all other variables will have decreasing lower scores. The importance score measures a variable's ability to perform in a specific tree of a specific size either as a primary splitter or as a ‘surrogate’ splitter. The relative importance ranking of variables tends to change dramatically when comparing trees of substantially different sizes. Therefore, the importance scores (rankings) are strictly relative to a given tree structure and should not be interpreted as the absolute information value of a variable.

The TREE command in SPSS [15] was used to generate the classification trees showing the classification rules generated through recursive partitioning and relative variable importance.

### Ethical clearance

The study received ethical clearance from the Ifakara Health Institute ethical review board (IHI/IRB/No.A67-2009) and national ethical clearance after having met the criteria for ethical considerations.

## Results

### Description of patient characteristics by health facility ownership

A total of 1,470 patients attended the outpatients department (OPD) of health facilities, most of them (1,116, 76%) at 18 publicly owned facilities and the rest at 12 private facilities (Table 2). More than half (53.5%) of the patients were less than five years old and 53% were females. The majority (71%) of patients attended OPD of a peripheral health facility while just 2.1% that of a hospital. Laboratory diagnosis was done in more than half of the patients (54%), while the rest were presumptively treated by a doctor and/or other clinical personnel. Laboratory tests were more commonly used in private (83%) than in public sector (45%) health facilities. Overall, the average number of drugs prescribed per encounter was 2.3 (range: 1–6) per patient, with private sector patients receiving more drugs than those in public (2.5 vs. 2.2). Only 15% of the patients were prescribed only one drug while 7% received more than three drugs on one visit (Table 2).

**Table 2:** Patient characteristics by health facility ownership

	**Public n = 1,116 (76%)**		**Private n = 354 (24%)**		**Total n = 1,470**	
	**N**	**%**	**N**	**%**	**N**	**%**
Gender						
Male	507	45.4	175	49.4	682	46.4
Female	609	54.6	179	50.6	788	53.6
Age groups						
Less than 5	597	53.5	104	29.4	701	47.7
5 to 14	189	16.9	56	15.8	245	16.7
15 +	330	29.6	194	54.8	524	35.6
Health facility type						
Dispensary	713	63.9	323	91.2	1036	70.5
Health centre	403	36.1	0	0	403	27.4
Hospital	0	0	31	8.8	31	2.1
Diagnosis mode						
Laboratory confirmation	499	44.7	295	83.3	794	54.0
Presumptive	386	34.6	36	10.2	422	28.7
Missing	231	20.7	23	6.5	254	17.3
Total number of drugs taken on this visit; *mean (95%CI)*	2.2(2.1-2.3)		2.5(2.4-2.6)		2.3(2.2-2.3)	
Number of drugs taken grouped						
One	178	16.0	42	11.9	220	15.0
Two	558	50.0	143	40.4	701	47.7
Three	311	27.9	121	34.2	432	29.4
More than three	59	5.3	44	12.4	103	7.0
Missing	10	0.9	4	1.1	14	0.9

**Figure 1:**
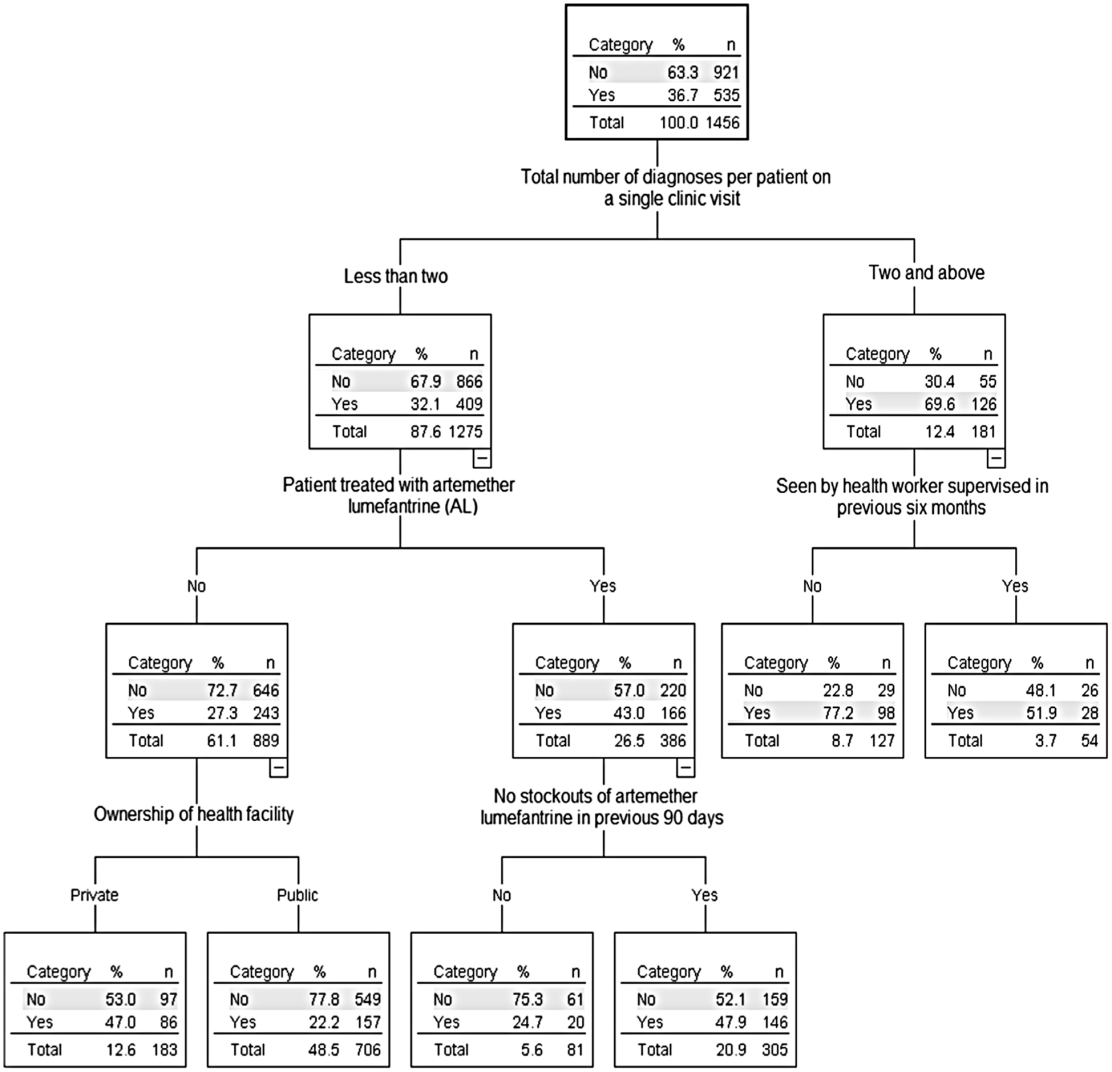
**Classification tree representing the predictors of polypharmacy prevalence. **Note: Inside the boxes (nodes); Yes = Greater or equal to three drugs (Polypharmacy), No = Less than three drugs.

Out of 1,470 patients interviewed, 14 did not have record of any medication taken. Of the remaining 1,456 patients who were prescribed at least one drug, 36.7% were prescribed three or more medicines (polypharmacy). Using the variables in Table 1 to fit the model, the most important predictor of polypharmacy was the total number of diagnosed illnesses at a single clinic visit – a patient-related factor (Table 3). This was followed by a facility related factor – ownership (private/public) with discriminatory power of 36.1%. Other factors were treating a patient with first-line drugs (AL) with a power of 27%, health worker age (power: 26%), a health worker being trained in IMCI with anti-malarial components, a facility not experiencing AL stock-out in the previous 90 days (health facility-related), health worker gender, health worker having been supervised in the previous six months (power: 11.5%), the remaining factors had<10% discriminatory power (Table 3). The difference in discriminatory power between the top predictor variable and the next most important predictor was substantial (100% *vs* 36.1%). Similar results are obtained by the classification tree model (Figure 1).

**Table 3:** Ranking of the predictors of polypharmacy by their overall discriminatory power

**Independent variable**	**Power**
Total number of diagnoses per patient on a single visit	100.0
Ownership of health facility (public/private)	36.1
Patient treated with artemether lumefantrine	26.8
Health worker age	26.0
Patient seen by health worker with IMCI training	16.1
No stockouts of artemether lumefantrine in previous 90 days	13.9
Health worker sex	12.1
Patient seen by health worker supervised within previous six months	11.5
Transmission season	4.8
Availability of any scale in the health facility	4.5
Patient seen by health worker with experience on caring patients	3.9
Supplies to administer oral medications	2.4
Type of health facility	1.0
Health workers' cadre	0.4
Any artemether lumefantrine available in health facility today	0.1

Patients diagnosed with more than one disease on a single attendance had higher chances of being prescribed three or more medicines: 69.6% (compared to 32.1% in those with one diagnosis). The next most important factor among patients with more than one diagnosis was supervision of health worker within the previous six months where polypharmacy occurred in 77.2% patients treated by an unsupervised health worker as compared to 52% in those treated by a supervised health worker (p-value<0.0001). For patients with one diagnosis, the next splitting factor was being treated with AL whereby more than three drugs were prescribed to 43% of patients treated with AL compared to 27.3% of those not treated with AL. Looking further at patients with one diagnosis and treated with AL, those treated in a facility that did not experience AL stock-out in the previous three months were more likely to be given more than three drugs (48%) compared to 25% of those served in clinics that experienced AL stock outs in the previous three months but this difference was not significant (p-value = 0.124). In patients with one diagnosis not treated with AL, ownership of facility was the next splitting factor where those served in privately owned facilities were more likely to receive three or more drugs compared to those in public facilities (47% *vs* 22.2%) and this was statistically significant (p-value = 0.003). Overall, the classification tree for polypharmacy (Figure 1) had a sensitivity of 67% and a specificity of 66%. Health worker’s age and training in Integrated Management of Childhood Illnesses (IMCI) may not have appeared in the classification tree as main splitters but are important variables as shown by their overall discriminatory power of 26% and 16.1% respectively (Table 3).

### Co-prescription of antibiotics with artemether-lumefantrine

Overall, 84.7% (n = 1,233) of the patients interviewed had more than one treatment with a median number of two prescribed medications per patient-clinic visit (range 1–6). Among the 508 (34.9%) patients treated with AL, the most commonly prescribed concomitant medications were analgesics (87.4%, n = 445), followed by antibiotics (41.6%, n = 212) and other medications (31.6%, n = 161). According to the overall discriminatory power from the classification tree analysis, patient age emerged as the strongest overall risk factor for co-prescription of AL with an antibiotic, closely followed by the season of the interview (power: 93%), mode of diagnosis (power: 90%), availability of national malaria guideline (power: 84.5%) (Table 4).

**Table 4:** Ranking the predictors of co-prescription of artemether-lumefantrine with antibiotics

**Independent variable**	**Power**
Age group of the patients	100.0
Transmission season	92.6
Mode of diagnosis	90.4
Possession of national malaria guideline	84.5
Possession of job aids	78.7
Patient seen by health worker with IMCI training	77.6
Patient seen by health worker supervised within previous six months	67.0
Patient seen by health worker with experience	40.2
Patient seen health worker who trained on use of new anti-malarial	36.8
Health worker sex	32.3
Possession of national malaria wall chart	26.9
Health worker age	18.9
Health workers' cadre	10.6
Patient seen by health worker with medical training	9.1
Ownership of health facility (public/private)	8.6

### Classification tree modelling for co-prescription of artemether-lumefantrine with antibiotics

**Figure 2:**
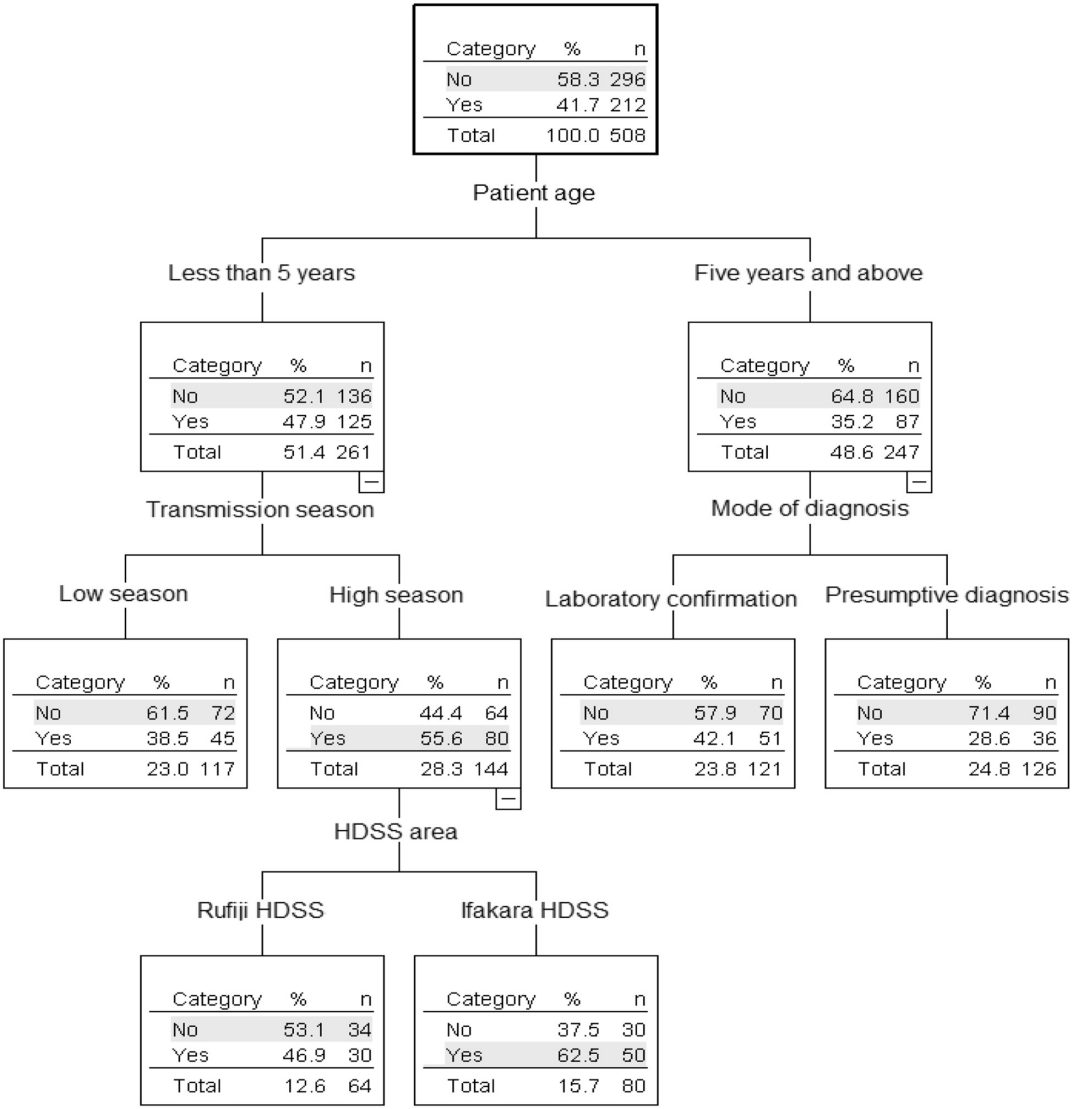
**Classification tree representing the predictors of co-prescription of artemether-lumefantrine (First line drug) with an antibiotic. **Note: Inside the boxes (nodes); Yes = Co-prescription of AL with antibiotics, No = No co-prescription of AL with antibiotics.

The classification tree partitioned the predictors according to the overall discriminatory power of variables (Figure 2). In modelling the co-prescription of AL with antibiotics, patient clinical status (number of diagnoses) was found to be very important and when included in the model it masked the effect of other variables. For that reason it was deliberately removed so as to examine and assess the importance of other factors which may not be as obvious. The classification tree for co-prescription of AL with antibiotics has the most important predictors as patient age, transmission season, mode of diagnosis and location of health facility in terms of HDSS. Co-prescription of AL with antibiotics was done for 41.7% of the patients visiting the clinics during the study period. This was common among patients aged less than five years, at 47.9% compared to 35.2% of those aged five years and above. For the older patients (≥5 year), this co-prescription was common in those treated after being tested for malaria (42.1%) compared to those presumptively treated (28.6%), though the difference was not statistically significant (P-value = 0.1471). In the under fives, co-prescription occurred more frequently during the high (55.6%) than in the low malaria transmission season (38.5%). The location of the facility was important whereby patients treated in the high malaria transmission season at a facility in the Ifakara HDSS catchment area were more likely to be co-prescribed with AL and an antibiotic (62.5%) than those served in Rufiji HDSS catchment area (46.9%) (P-value = 0.0086). Some variables did not appear as main splitters in the tree despite their overall high discriminatory power (Table 4). This is due to the fact that they are important at several stages of the classification building tree but never as important as the main splitter.

## Discussion

The first step to improve the rational use of drugs is to understand prescribing patterns. This paper demonstrates the application of classification tree analysis models a non-parametric modelling methodology to explore factors influencing drug prescription practices in health facilities of rural Tanzania. Classification trees are user friendly and easy to interpret and have been utilized to identify the main risk factors for malaria infection in Burundi and Vietnam [21,23]. In this analysis, the classification tree method revealed logical results of the relationships between the outcomes of interest (polypharmacy and co-prescription of AL with antibiotics) and the predictor variables.

While multinomial models reveal factors that predict the outcome in the whole population, classification tree analysis helps in detecting population segments that need specific attention in relation to the outcome. Segmenting populations supports decision makers in targeting their efforts to specific subgroups. It is important to note that this analysis does not support any claim of superiority of one methodology compared to the other.

This analysis demonstrated some real-life treatment practices at the facilities. It is common for most patients to report with more than one complaint, which compels the health worker to prescribe more than one medication for each identified illness. The IMCI strategy was introduced by WHO to reduce child morbidity and mortality. Indeed, treatment of childhood illness may also be complicated by the need to combine therapy for several conditions [25]. It is therefore not surprising that the total number of diagnoses was the most important predictor of polypharmacy as revealed by both its ranking in terms of importance and being a major splitter in the classification tree. A plausible explanation is that health workers are insecure about the diagnosis since in most cases the available laboratory services are unable to accurately determine the cause of illness. Therefore, to satisfy the patients, the health worker prescribes more drugs and then justifies this practice by diagnosing several pathological conditions.

Supervision of health workers is another important predictor of polypharmacy. Indeed, polypharmacy was more common among unsupervised health workers. It is worth noting that this study did not comprehensively explore the type of supervision, limiting the conclusions on the possible consequences of not having adequate supervision on prescription practices. Nonetheless, it would be advisable for district health authorities to include drug prescription practices during their routine supervision visits.

Polypharmacy is common in the private sector where individual motivation and incentives may have preponderance over the knowledge and skills of the providers. Health worker age, sex, being trained in IMICI and being supervised in previous six months were the health worker-related variables identified as the other important variables that explain polypharmacy. The observation that patients treated with AL in a clinic that did not experience stock-outs of artemether-lumefantrine are more likely to be prescribed several treatments is expected and could be due to the IMCI strategy, which recommends this practice, especially for children presenting with multiple symptoms.

In general, presumptive diagnosis was common in public facilities while laboratory results were more used in privately owned facilities. In SSA, it is common practice not to use the test result when treating fever cases [26]. The practice of presumptive treatment for malaria has been and is still being practiced in several health facilities, both in rural- and urban-based centres, because the syndromic treatment for febrile illnesses has been standard practice for long time, and clinicians mistrust the laboratory results due to poor quality of the laboratory tests. Patients with a negative malaria test are still treated with an anti-malarial on the grounds that signs and symptoms are compatible with the diagnosis of malaria. This continues even after the introduction of malaria rapid diagnostic tests. [26]

The recommended first-line anti-malarial drug (AL) was more commonly used in public than in private facilities as the former are supplied with essential drugs directly by the central pharmacy. This may change with the introduction of the Affordable Medicine Facility for malaria (AMFm) strategy in Tanzania whose approach is to supply subsidized AL to the private sector [27]. It will therefore be interesting to look at how these changes in the health system will affect the prescription patterns in Tanzania and other African countries over time.

There was a high level of co-prescription of antibiotics with AL, particularly in children less than five years living around the Ifakara area. A study in Ghana conducted predominantly in government facilities in an urban setting showed that 30.8% of patients were receiving at least one antibiotic in addition to the recommended anti-malarial [28]. Co-prescription with antibiotics is a life saving practice and commonly practiced in health facilities in sub-Saharan Africa since patients can present with multiple illnesses at a single clinic visit. This is why it was promoted under the IMCI strategy. However, this has implications for the patient’s safety as it may increase the risk of drug-drug interactions (D-DI), therapeutic failure, drug resistance and adverse events [29]. If this practice of co-prescription of drugs, which is common in rural health facilities, is not addressed, it may cause a major problem as the risk of adverse drug events (ADE) increases with an increasing number of medicines prescribed [30-33].

Classification tree analysis models are useful in expressing relationships between variables since they do not need to be linear or additive and the possible interactions do not need to be pre-specified or of a particular multiplicative form. Results are presented in the form of a decision tree, a different approach than the standard statistical analysis. The results highlight areas that merit further attention and can act as a guide for further epidemiological and hypothesis-driven research. The classification trees provide a more flexible relationship between variables; missing values of the covariates, multi-colinearity and outliers are taken care of in an intuitively and correct manner [19]. This methodology has proven its usefulness and adequacy in other areas and contexts, for example the bee colony collapse disorder, bovine spongiform encephalopathy and analysis of urban farming systems in central Africa [17,19,20]. In malaria, this method has been used for ranking highland malaria risk factors in Burundi and in Vietnam [21,23]. However, it has a limitation of not providing p-values and standard deviations as in familiar parametric methods. Another limitation is that confounding values make classification tasks more difficult. Although this decreases true positive rates and accuracies, the constructed classification trees are valuable. The benefit of the trees is that they simulate more the real life situation with patients who have confounding attributes. Future work should be aimed at finding different ways to handle confounding values in the reasoning process. Another advantage is that the importance of the variable can still be seen in the variable relative importance.

A variable may be ranked among the top ones for the discriminatory power but may not appear as an important splitter in the classification tree, e g, training in IMCI with anti-malarial component. This happens because it is an important surrogate but not a major splitter. The ranking by overall discriminatory power is determined by the sum across all nodes in the tree of the improvement score that the predictor has when it acts as a primary or a surrogate splitter. Consequently, a health worker having IMCI training with anti-malarial component enters the tree as the top surrogate splitter in many nodes but never as primary splitter.

Initiatives like the INESS Phase IV platform, working within communities through the HDSS system should continue to evaluate the effect of provider practice on new and old products and may be extended to other therapeutic areas such as ARVs, anti-TBs, antibiotics and vaccines. Inclusion of the private sector, e g, private pharmacies, retail shops, mobile drug sellers and even traditional herbalists will provide public health managers with more evidence on which to base their decisions.

## Conclusions

The classification tree analysis approach can be used to classify prescription patterns using health facility information. This procedure offers an opportunity to examine alternative methods of identifying predictors of prescription patterns that might assist decision makers to improve targeted service provision factors. This study has demonstrated that polypharmacy is mainly associated with multiple diagnoses while co-prescription of AL with antibiotics is mainly associated with patient age. Although these are considered life saving practices, they expose patients to risks of adverse drug reactions. Bacterial antibiotic resistance should be looked as a public health emergency and the two competing causes of increased bacterial resistance are irrational antibiotic use and availability of poor quality antibiotics. Drug prescription practices may improve by introducing targeted interventions such as regular supervision of health care providers in both public and private health facilities.

## Competing interests

The authors declare that they have no competing interests.

## Authors’ contributions

DK drafted the manuscript in consultation with NS; IM, BA, RK, AD, FB and DK contributed to study coordination and review of subsequent manuscript; MS, MN supervised data entry, cleaning, preliminary analysis and review of subsequent manuscript. DK conducted the data mining analyses in consultation with NS; DK, NS, JM, UDA and AD reviewed the manuscript. All authors read and approved the final manuscript.

## References

[B1] Trostle J Inappropriate distribution of medicines by professionals in developing countries Soc Sci Med 1996 42 1117 1120 10.1016/0277-9536(95)00384-3 8737428

[B2] World Health Organization Report of the conference of Experts The Rational Use of Drugs 1987 WHO, Nairobi 25 29

[B3] World Health Organization Medicines use in primary care in developing and transitional countries. Fact Book summarizing results from studies reported between 1990 and 2006 2009 WHO, Geneva WHO/EMP/MAR/2009.3

[B4] Frischer M Heatlie H Norwood J Bashford J Millson D Chapman S Trends in antibiotic prescribing and associated indications in primary care from 1993 to 1997 J Public Health Med 2001 23 69 73 10.1093/pubmed/23.1.69 11315698

[B5] McManus P Hammond ML Whicker SD Primrose JG Mant A Fairall SR Antibiotic use in the Australian community Med J Aust 1997 167 124 127 9269265 10.5694/j.1326-5377.1997.tb138809.x

[B6] Ministry of Health and Social Welfare URoT National Guidelines for Diagnosis and Treatment of Malaria control series 11 2006 National Malaria Control Program, Dar es Salaam

[B7] World Health Organization WHO guidelines for the treatment of malaria 2006 WHO, Geneva 1108/HTM/MAL/2006

[B8] Zurovac D Rowe AK Ochola SA Noor AM Midia B English M Snow RW Predictors of the quality of health worker treatment practices for uncomplicated malaria at government health facilities in Kenya Int J Epidemiol 2004 33 1080 1091 10.1093/ije/dyh253 15256523

[B9] Brugha R Chandramohan D Zwi A Management of malaria –working with the private sector Trop Med Int Health 1999 4 402 406 10.1046/j.1365-3156.1999.00411.x 10402978

[B10] Skarbinski J Ouma PO Causer LM Kariuki SK Barnwell JW Alaii JA de Oliveira AM Zurovac D Larson BA Snow RW Rowe AK Laserson KF Akhwale WS Slutsker L Hamel MJ Effect of malaria rapid diagnostic tests on the management of uncomplicated malaria with artemether-lumefantrine in Kenya: a cluster randomized trial Am J Trop Med Hyg 2009 80 919 926 19478249

[B11] Rufiji DSS Tanzania Ministry of Health Tanzania Essential Health Interventions Project. Adult Morbidity and Mortality Project- Indepth Monograph: Vol 1 Part Chttp://www.indepth-network.org/dss_site_profiles/rufiji.pdf accessed on 12th Aug, 2012

[B12] Ifakara HDSS Tanzania Accessed at http://www.indepth-network.org/leadership/IFAKARA%20HDSS.pdf on 12th Aug, 2012

[B13] Lauritsen JM Bruus M EpiData (version 3). A comprehensive tool for validated entry and documentation of data 2003 The EpiData Association, Odense, Denmark

[B14] StataCorp LP STATA version 11. 2012, College Station 2012 STATA Corporation, Texas, USA

[B15] IBM SPSS software SPSS for Windows version 16 2007 SPSS Inc, Chicago

[B16] Speybroeck N Classification and Regression trees Int J Publ Heath 2012 57 243 246 10.1007/s00038-011-0315-z 22015650

[B17] Saegerman C Speybroeck N Roels S Vanopdenbosch E Thiry E Berkvens D Decision support tools for clinical diagnosis of disease in cows with suspected bovine spongiform encephalopathy J Clin Microbiol 2004 42 172 178 10.1128/JCM.42.1.172-178.2004 14715749 PMC321688

[B18] Breiman L Friedman JH Olsen RA Stone CJ Classification and regression trees 1984 The Wadsworth statistics/probability Series, Belmont, California

[B19] Speybroeck N Berkvens D Mfoukou-Ntsakala A Aerts M Hens N Van Huylebroeck G Thys E Classification trees versus multinomial models in the analysis of urban farming systems in Central Africa Agric Systems 2004 80 133 149 10.1016/j.agsy.2003.06.006

[B20] van Engelsdorp D Speybroeck N Jay DE Nguyen B Mullin C Frazier M Frazier J Cox-Foster D Chen Y Tarpy D Haubruge E Pettis J Saegerman C Weighing risk factors associated with bee colony collapse disorder by classification and regression tree analysis J Econ Entomol 2010 103 1517 1523 10.1603/EC09429 21061948

[B21] Thang ND Erhart A Speybroeck N Hung LX Thuan LK Cong TH Pham VK Coosemans M D’Alessandro U Malaria in central Vietnam: analysis of risk factors by multivariate analysis and classification tree models Malar J 2008 7 28 10.1186/1475-2875-7-28 18234102 PMC2267804

[B22] Zhang H Singer B Recursive Partitioning in the Health Sciences 1999 Springer, New York

[B23] Protopopoff N Van Bortel W Speybroeck N Van Geertruyden J-P Baza D D’Alessandro U Coosemans M Ranking malaria risk factors to guide malaria control efforts in African Highlands PLoS One 2009 4 e8022 10.1371/journal.pone.0008022 19946627 PMC2778131

[B24] McCarthy FD Wolf H Wu Y The growth costs of malaria 2000 National Bureau of Economic Research, 2000 Working Paper 7541, Cambridge, MA, USA

[B25] WHO/UNICEF Integrated Management of Childhood Illness IMCI Information package 1999 WHO/CHS/CAH/98

[B26] Rennie W Lugo L Rosser E Harvey SA Willingness to use and pay for a new malaria diagnostic test for children under 5: Results from Benin, Peru, and Tanzania 2009 Center for Human Services, Bethesda, MD

[B27] The Affordable Medicines Facility for Malaria (AMFm) 2012 http://rbm.who.int/psm/amfm.html last accessed on 12th Aug 2012

[B28] Dodoo A Fogg C Asiimwe A Nartey E Kodua A Tenkorang O Ofori-Adjei D Pattern of drug utilization for treatment of uncomplicated malaria in urban Ghana following national treatment policy change to artemisinin-combination therapy Malar J 2009 8 2 10.1186/1475-2875-8-2 19123926 PMC2647941

[B29] Mino-León D Galván-Plata ME Doubova SV Flores-Hernandez S Reyes-Morales H A pharmacoepidemiological study of potential drug interactions and their determinant factors in hospitalized patients Rev Invest Clin 2011 63 170 178 21717723

[B30] Chrischilles EA VanGilder R Wright K Kelly M Wallace RB Inappropriate medication use as a risk factor for self-reported adverse drug effects in older adults J Am Geriatr Soc 2009 57 1000 1006 10.1111/j.1532-5415.2009.02269.x 19507293

[B31] Fu AZ Liu GG Christensen DB Inappropriate medication use and health outcomes in the elderly J Am Geriatr Soc 2004 52 1934 1939 10.1111/j.1532-5415.2004.52522.x 15507075

[B32] World Health Organization Introduction to drug utilization research 2003 WHO, Geneva

[B33] Laroche ML Charmes JP Nouaille Y Picard N Merle L Is inappropriate medication use a major cause of adverse drug reactions in the elderly? Br J Clin Pharmacol 2007 63 177 186 10.1111/j.1365-2125.2006.02831.x 17166186 PMC2000580

